# Effectiveness and cost-effectiveness of a tailored text-message programme (MiQuit) for smoking cessation in pregnancy: study protocol for a randomised controlled trial (RCT) and meta-analysis

**DOI:** 10.1186/s13063-019-3341-4

**Published:** 2019-05-22

**Authors:** Rachel Whitemore, Jo Leonardi-Bee, Felix Naughton, Stephen Sutton, Sue Cooper, Steve Parrott, Catherine Hewitt, Miranda Clark, Michael Ussher, Matthew Jones, David Torgerson, Tim Coleman

**Affiliations:** 1 Division of Primary Care, University of Nottingham, Nottingham, NG7 2RD UK; 2 Division of Epidemiology and Public Health, University of Nottingham, City Hospital, Nottingham, NG5 1PB UK; 3 School of Health Sciences, University of East Anglia, Norwich, NR4 7TJ UK; 4 Institute of Public Health, University of Cambridge, Cambridge, CB2 0SR UK; 5 Department of Health Sciences, Seebohm Rowntree Building, University of York, Heslington, York, YO10 5DD UK; 6 Population Health Research Institute, St. George’s, University of London, London, SW17 0RE UK; 7 UK Centre for Tobacco and Alcohol Studies, Nottingham, UK; 8 Institute for Social Marketing and Health, University of Stirling, Stirling, FK9 4LJ UK

**Keywords:** Smoking cessation, Pregnancy, Self-help, Randomised controlled trial, Protocol

## Abstract

**Background:**

Smoking in pregnancy is a major international public health problem. Self-help support (SHS) increases the likelihood of women stopping smoking in pregnancy and delivering this kind of support by text message could be a cost-effective way to deliver SHS to pregnant women who smoke. SHS delivered by text message helps non-pregnant smokers to stop but the currently available message programmes are not appropriate for use in pregnancy.

A randomised controlled trial (RCT) has demonstrated the feasibility and acceptability of using a programme called ‘MiQuit’ to text SHS support to pregnant women who smoke. Another pilot RCT has shown that it would be feasible to run a larger, multi-centre trial within the UK National Health Service (NHS). The aim of this third RCT is to complete MiQuit’s evaluation, demonstrating whether or not this is efficacious for smoking cessation in pregnancy.

**Methods/design:**

This is a multi-centre, parallel-group RCT. Pregnant women aged over 16 years, of less than 25 weeks’ gestation who smoke one or more daily cigarettes but smoked at least five daily cigarettes before pregnancy and who understand written English and are being identified in 24 English antenatal care hospitals. Participants are randomised to control or intervention groups in a 1:1 ratio stratified by gestation (< 16 weeks versus ≥ 16 weeks). All participants receive a leaflet on stopping smoking during pregnancy; they are also able to access standard NHS smoking cessation support. Intervention group women also receive the 12-week MiQuit programme of tailored, interactive text message, and self-help cessation support. Women are followed up by telephone at 4 weeks after randomisation and 36 weeks’ gestation. The RCT will recruit 692 women (346 per group), enabling a 95% confidence interval for the difference in quit rates to be estimated within ± 3%. To determine whether or not MiQuit helps pregnant smokers to stop, intervention group quit rates from this trial will be combined with those from the two earlier trials in a Trial Sequential Analysis (TSA) meta-analysis to derive a pooled efficacy estimate.

**Discussion:**

If effective, MiQuit will be a cheap, cost-effective method to help pregnant women to stop smoking.

**Trial registration:**

ClinicalTrials.gov, ID: NCT03231553. Registered on 20 July 2017.

**Electronic supplementary material:**

The online version of this article (10.1186/s13063-019-3341-4) contains supplementary material, which is available to authorized users.

## Background

As well as causing cancer, smoking is strongly associated with increased pregnancy-specific risks of miscarriage, stillbirth, prematurity, low birth weight, perinatal morbidity and mortality, neo-natal and sudden infant death, [1] poorer infant cognition and adverse infant behavioural outcomes [2, 3]. Smoking in pregnancy is expensive to health care services; in the UK in 2010 the annual smoking-attributable maternal and infant health care costs were estimated at up to £87.5 million [4]. In high-income countries just over 10 to 25% of pregnant women smoke, with highest rates seen amongst younger, socially disadvantaged women, [5–9] and rates are also increasing in developing countries [10]. In the UK in 2016/17, 10.5% of pregnant women were known to be smokers at time of delivery [9]. However, pregnancy is the life event which most motivates smokers’ cessation attempts and over 50% of pregnant smokers try stopping [8], hence smoking cessation support offered in pregnancy is likely to be especially beneficial. In pregnancy, there is strong efficacy evidence for using either face-to-face [11], or ‘self-help’ [12] stop-smoking support and some evidence that financial incentives [13] are effective as adjunctive support. Although nicotine replacement therapy (NRT) is widely used by UK pregnant smokers [14, 15], this has, at best, borderline efficacy [16]. Poor adherence to NRT [17] and accelerated nicotine metabolism in pregnancy [18, 19] may explain why NRT works well outside of [20], but not during, pregnancy [16].

Self-help support almost doubles the odds of cessation in late pregnancy (OR 1.83, 95% CI 1.23 to 2.73) [12]. However, self-help programmes that have been shown to help pregnant smokers to quit were all developed before easily accessible digital technologies became widely available [12]. Self-help text-message smoking-cessation programmes are highly acceptable; those trialled with *non-pregnant smokers* in the US [21, 22] and UK [23] have demonstrated efficacy. Unfortunately, neither programme is appropriate in pregnancy as they make no mention of pregnancy, which, for most pregnant smokers, is the very reason they try quitting; consequently many pregnant smokers would likely find much of the advice from these programmes irrelevant and ignore it. Additionally, some of these programmes’ recommendations could be harmful in pregnancy. For example, in pregnancy, advice about NRT, keeping fit and weight gain after quitting are necessarily quite different from advice given to those who are not pregnant. Although there is a self-help, cessation-orientated text programme available for pregnant smokers in the US [24] and various self-help support ‘apps’ aimed at encouraging pregnant smokers to quit, these have not yet been definitively evaluated.

To remedy the lack of acceptable self-help cessation support for pregnant smokers, we developed MiQuit, a text-message, smoking cessation self-help support programme for pregnant smokers. MiQuit advice is orientated to pregnancy and is highly tailored to the woman’s motivation to quit, and her attitudes, beliefs and behaviours related to smoking. We have already evaluated MiQuit in two randomised controlled trials (RCTs). The first trial (*n* = 207) demonstrated *acceptability*; 87% of recipients read every text and only 9% terminated the programme early [25]. Estimated efficacy was encouraging; 12 weeks after randomisation, biochemically validated abstinence rates in MiQuit and control groups were 12.5 and 7.8%, respectively (OR 1.68, 95% CI 0.90 to 3.16) [25]. Subsequently, we made a few minor modifications to MiQuit and tested this in a second RCT which demonstrated the *feasibility of recruiting from UK National Health Service (NHS) settings to a multi-centre RCT* [26]; we recruited 407 participants from 16 trial centres in 7 months (approximately 3.5 women/centre/month). Study retention was high with only 10 withdrawals (three revoked consent, seven withdrew after fetal deaths). Again, estimated efficacy was encouraging; in MiQuit and control groups, prolonged abstinence from smoking, validated in late pregnancy was 5.4 and 2.0%, respectively (OR 2.70, 95% CI 0.93–9.35).

Although smoking cessation rates of this size may appear small, the substantial harm caused by smoking means that, at a population-level, such a difference would be clinically important [27]. Hence, if MiQuit were to have demonstrable efficacy, it could be integrated into routine clinical practice with beneficial effects for women who smoke in pregnancy and their infants. This trial is designed to produce sufficient data such that a definitive assessment of MiQuit efficacy will be possible.

### Primary objective

The primary objective of this trial is to assess the efficacy of the MiQuit system, when offered in addition to standard behavioural support for smoking cessation in pregnancy, by synthesising findings from this RCT with those from the two earlier MiQuit RCTs using Trial Sequential Analysis (TSA) meta-analysis.

### Secondary objectives

The secondary objectives for this trial are as follows:

1. To compare validated rates of prolonged smoking cessation between 4 weeks after enrolment and 36 weeks’ gestation (late pregnancy) between MiQuit and control groups
2. To compare rates of adverse pregnancy outcomes between MiQuit and control groups
3. To estimate the incremental cost-effectiveness of MiQuit when added to the usual smoking cessation care

## Methods/design

### Trial design

This study is a multi-centre, two-arm, parallel-group, single-blind, individually randomised controlled trial testing the effectiveness of the MiQuit text-message smoking-cessation support service in pregnant women.

### Study setting

Participants will be recruited from hospital antenatal clinics in England.

### Eligibility criteria

To be eligible for the trial, participants must (1) be less than 25 weeks pregnant, (2) have smoked at least five cigarettes per day pre-pregnancy and have continued to smoke at least one cigarette on a typical day during pregnancy, (3) be aged 16 years or over, (4) agree to accept information to assist cessation, (5) have their own or have primary use of a mobile phone, (6) be familiar with sending and receiving text messages, (7) be able to understand written English (text messages are in English only) and consent issues explained in English and (8) be able to give informed consent.

If women express an interest in stopping smoking but are not eligible to join the study, they will be sign-posted to the local stop-smoking services, as part of routine clinical practice, where these are available.

Participants should not be enrolled in another text service to assist smoking cessation, be enrolled in any other smoking cessation studies, or have already participated in another MiQuit study in a previous pregnancy.

### Control

Control group participants will receive a standard NHS leaflet which provides information and advice on stopping smoking, in addition to their usual NHS antenatal care.

### Intervention

All intervention group participants will receive the same standard NHS leaflet as control participants giving information and advice on stopping smoking and usual NHS antenatal care.

Additionally, they will receive MiQuit. MiQuit is a 12-week, automated, responsive, text-message, self-help support programme which sends tailored smoking cessation support and advice to participants’ mobile phones. MiQuit system content is tailored to 12 participant characteristics collected at baseline. This can be further tailored to changes in smoking status, collected via texts sent by the participant to the MiQuit service, at two time points during the programme. In addition, participants can text in a quit date and receive additional support tailored around this date. Participants can increase or decrease the frequency of support that they receive at any time by texting the keywords MORE or LESS. The support includes motivational messages, advice about preparing for a quit attempt, how to manage cravings and withdrawal, dealing with trigger situations, information about how smoking affects babies, and general encouragement. Depending on how participants use the system they will receive between 69 and 121 texts over the 12 weeks (0.8 to 1.5 texts per day). Participants can also request on-demand instant text messages to further support or distract them by texting one of three keywords to the MiQuit system (QUIZ, HELP and SLIP). Any text-message replies sent to MiQuit by participants are charged at usual network rates or included in their text allowance, but texts that they receive from MiQuit are free. All likely costs to participants will be made clear in the Participant Information Leaflet (PIL).

### Adherence

Participants allocated to receive the MiQuit intervention will receive a complete programme of text messages; however, they can actively opt out of receiving more messages by texting STOP to MiQuit at any time point, thereby discontinuing their treatment. Consequently, adherence could be assessed by considering whether or not participants received a full programme or the length of any partial programme that they received prior to terminating this.

### Outcomes

#### Primary outcome

The primary outcome for this trial will be the self-reported smoking abstinence from 4 weeks after enrolment until 36 weeks’ gestation, reported and biochemically validated at 36 weeks by exhaled carbon monoxide (CO) and/or saliva cotinine and anabasine estimation; there will be no ‘grace period’ [28]. Participants who report smoking no more than five cigarettes in total between the start of their quit attempt (within 4 weeks of randomisation) and late pregnancy will be considered to have quit smoking [29]. Data for this outcome will be combined with that from previous trials in a TSA meta-analysis – *as described in the ‘Statistical methods’ section below*).

#### Secondary outcomes

Smoking outcomes

1. Seven-day abstinence self-reported at 4 weeks; self-reported and biochemically validated 7-day abstinence at 36 weeks

Other outcomes for the trial are as follows:

2. Participants’ reported use of stop-smoking services and use of NHS care whilst involved in the trial

3. *Pregnancy outcomes*: birth weight; gestation at birth; or maternal or fetal death (i.e. stillbirth or miscarriage); maternal/infant hospital admissions

4. Economic measures: EuroQol-5D-5L Level Questionnaire (EQ-5D-5 L), costs of providing the text-message service (staff time, costs of maintaining text-messaging software), costs of the usual care intervention, and wider costs of health care admissions for mother and infant

### Assignment of interventions: allocation and blinding

Enrolment and randomisation will be conducted once the participant’s baseline data has been submitted onto a secure online database by local research site staff. As randomisation will be via the Internet, allocations will be concealed from both local research site staff and participants. Randomisation will use a computer-generated pseudo-random code with random permuted blocks of randomly varying size created by the York Trials Unit (YTU) in accordance with their standard operating procedure and held on a secure server. The randomisation will be stratified by gestation (< 16 weeks versus ≥ 16 weeks).

Following randomisation, an automated email will be sent from the online database to the Nottingham Trial Office which will inform un-blinded central administrators, who are not involved in participant follow-up, of the allocation; a study information pack will then be sent to participants providing them with further information on their allocation within the trial, after which participants will no longer be blinded to their allocation. As far as possible, Trial Office staff who are involved in conducting the follow-ups will remain blind to treatment allocations; however, as participants are not blinded it is possible that they may unintentionally reveal their allocation during a follow-up telephone call (see later under ‘Visit 3’). The statistician conducting analyses will have no contact with participants and will be blinded to treatment allocations.

### Identification and recruitment of participants

Information about the trial will be displayed in relevant clinical areas and adverts in clinic environments may also be used. Women will be identified in early pregnancy, as they attend hospitals for antenatal screening (ultrasound) appointments, either by questionnaire or from medical records. *Questionnaire*: a member of the NHS care team (e.g. receptionist or local research staff) will hand all pregnant women attending clinics a self-complete screening questionnaire with an explanatory PIL attached. The PIL provides a detailed explanation of the trial in order to prevent feeding participants’ expectations about receiving any particular intervention. The screening questionnaire will identify smokers, and those who indicate that they would like to receive self-help cessation support as part of a research study will be asked to provide contact details, which can be shared with the research team. *Medical records*: alternatively, a member of the NHS care team will inspect clinic attenders’ records, identify those who are potentially eligible, and provide the pregnant smoker with a PIL.

This method of recruitment only applies to those attending clinic and there are no mechanisms to be used through which women will be recruited to the trial if they do not attend clinic. In addition, medical records will be examined purely to identify pregnant women who are potentially eligible because they are recorded as smokers; aged 16 years or over; and able to understand written English. This is so that as many eligible participants as possible can be approached and invited to take part in the trial. As there have been few previous similar trials, it is not certain how a researcher could predict propensity to consent to trial enrolment from medical records.

There will be two recruitment methods for eligible and interested pregnant smokers who have read and understood the PIL. These are as follows:

1. *Via staff in the NHS Trust*: In centres where local research staff are available, trial enrolment will be offered to all eligible, interested women. Consent will be obtained in person after women have completed the screening questionnaire, demonstrated that they have read and understood the PIL and confirmed that relevant aspects of trial procedures have been fully explained. Where possible, this will be done whilst women are waiting in clinic so that written informed consent can be obtained. Should the patient need to leave the clinic before consent can be obtained, a local research staff member may call and consent the patient over the telephone as for Method 2 (below). These patients will be provided with a PIL to take home and read and will be asked for a convenient time to be contacted by telephone. During the call, the trial will be fully explained and consent provided verbally. If necessary, some women may be called by a member of the Nottingham trial team instead; in these instances, consent will be obtained as for Method 2, below
2. *Via Nottingham Trial Office*: In centres where local research staff are not available, depending on other available resources, the same participant identification methods may be used. Members of the local NHS care team will oversee the collation of screening questionnaires within clinic and ensure that eligible and interested women’s contact details have been provided. The women would be provided with a PIL to take home and read. The contact details will then be sent to the Nottingham Trial Office, via either telephone call, email, post or shared via a secure, online means; a member of the Nottingham trial team would then telephone potential participants, ensuring that all aspects pertaining to trial participation are verbally addressed prior to obtaining consent

Reasons why potential participants are not recruited will be collected via the screening and enrolment logs.

NHS Hospital Trusts across England, with varying smoking rates amongst pregnant women, will be used as recruitment sites.

### Withdrawal of participants from intervention or assessments

Participants may be withdrawn from the trial either at their own request or at the discretion of the investigator. Participants who experience miscarriage or fetal death may be advised to withdraw from the trial. A list of participants due to be followed up will be sent to site staff prior to their scheduled visit. This will assist in identifying any participants whose medical records show them to have experienced an event which might make follow-up distressing, e.g. miscarriage. This approach was used in the latter stages of the pilot trial [30] and it was found that most participants were agreeable to discussion of smoking status, irrespective of whether miscarriage had occurred, provided that the researcher conducting follow-up was appropriately sensitive.

### Participant timeline and data collection

**Fig. 1:**
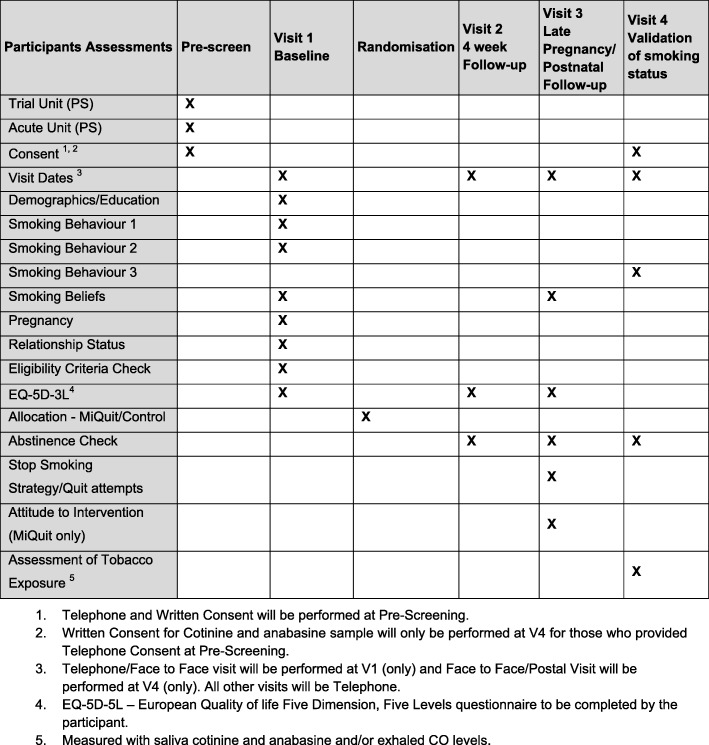
Schedule of participant assessments

**Fig. 2:**
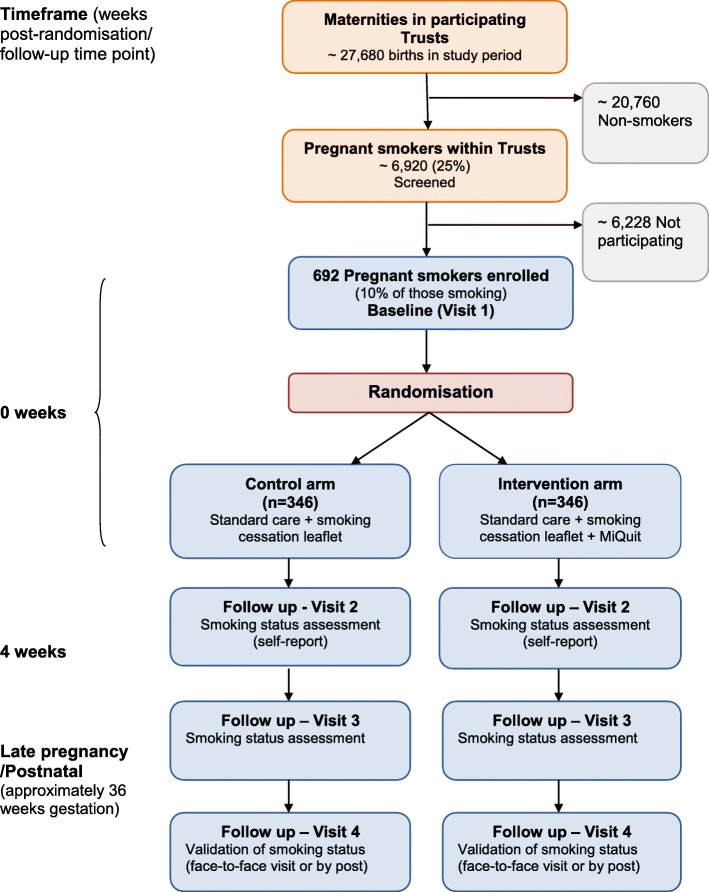
Study design, measures and estimated sample

Figure 1 describes the participant assessments at each stage. Figure 2 provides an overview of the study design and measurement time points.

### Pre-screen

All potential participants will be identified via a brief screening questionnaire, accompanied by a PIL.

Recruitment Method 1: For recruitment by this method local research staff working within the participating acute NHS Trust will explain all aspects of the trial and will counter-sign the consent form. The participant will be able to provide either written informed consent (face-to-face) or verbal informed consent (telephone).

Recruitment Method 2: For recruitment by this method a member of the Nottingham trial study team will be required to complete the consent form to indicate that all relevant issues have been addressed and the patient is eligible to be recruited into the trial. The study team member will sign the participant’s consent form and mark it as being completed via telephone.

Participants will be asked to provide written informed consent for the potential collection of an exhaled breath and/or saliva sample after their 36-week gestation follow-up by a member of the local research staff either during a hospital visit or by a visit to their home. However, they will be informed that samples would only be required if they were to report 7-day abstinence from smoking at this time point.

All participants will be provided with clear information about how to withdraw consent using a Freepost postcard (provided to all participants in their study information pack), text, email or telephone. In a similar trial in which we provided similar methods to facilitate informed consent, less than 60 participants (from over 2500) withdrew consent after enrolment [31].

### Visit 1 – Baseline

After giving informed consent, participants will be asked to complete a baseline questionnaire in person or via telephone with local research staff (*recruitment Method 1*) or with a Nottingham trial team member by telephone (*recruitment Method 2*).

The researcher will enter the participant details on the electronic database and randomise them to intervention (MiQuit) or control using the York Trials Unit’s web-based service. The researcher will remain blind to participants’ treatment allocations. For participants in either trial arm, a specific information leaflet describing only details of procedures employed within that arm will be generated only after randomisation. This is to minimise the number of participants who may wish to withdraw due to dissatisfaction with their treatment allocation and is and sent to them by a non-blinded member of the trial team who is not involved in follow-up. All participants will also be sent a standard NHS leaflet giving advice on stopping smoking during pregnancy, contact details for participants who have questions about trial involvement and information on how to withdraw if they change their mind. The PIL, information sheets and consent form will be available in English only as understanding of English is required to receive the MiQuit intervention.

Follow-up contacts will use a blend of postal, telephone, email/web and SMS text-messaging reminders to elicit maximal response rates.

### Visit 2 – 4-week follow-up

*At 4 weeks after randomisation*, participants will be contacted via telephone in order to assess smoking status, and obtain quality of life information (EQ-5D-5 L) in the period since randomisation. This visit will, in general, be performed via telephone by a member of the study team at the co-ordinating centre, blind to treatment allocation. However, should the study team be unable to contact the participant by telephone other methods will be used in an attempt to elicit the best response. These include; posting a short questionnaire with explanatory letter and pre-paid return envelope, and/or emailing a link which allows web-based completion.

Whilst it is theoretically possible that members of the Nottingham Trial Office team who speak to participants at the 4-week follow-up could become aware of participants’ treatment group and hence un-blinded for data collection at the late-pregnancy follow-up (Visit 3), we found that this was not an issue in the pilot study due to the time lapse between follow-up points. Additionally, as in the pilot trial, a number of different researchers will be involved in collecting follow-up data and, therefore, different researchers within the trial team may be collecting an individual’s data at Visit 2 (4 weeks after randomisation) and Visit 3 (36-week gestation) follow-ups.

### Visit 3 – Late pregnancy/postnatal follow-up

*At 36 weeks’ gestation*, participants will be asked to complete a questionnaire (data to be collected up to 10 weeks after estimated delivery date will be acceptable for use). This will include quality of life information (EQ-5D-5L), number of quit attempts lasting more than 24 h, measures of smoking behaviour, attitudinal/behavioural information, and use of NHS smoking cessation support and participants’ ratings of the intervention (MiQuit arm only). As for the 4-week visit, this visit will be performed mainly via telephone by a member of the study team at the co-ordinating centre, blind to treatment allocation. As for the 4-week visit, previously described alternative methods will be employed to elicit the best response rate.

It is recognised that asking about the intervention at follow-up will result in the researcher who conducts the late-pregnancy follow-up becoming un-blinded. To ensure that this has minimal impact on the follow-up data obtained, the participants will be asked items about the intervention at the end of the interview/questionnaire starting with the question: ‘*Did you receive any stop-smoking text messages from the study team*?’

At approximately 36 weeks’ gestation (Visit 3), participants will be asked basic questions about their recall of and use of advice in texts sent; however, this is a process measure to check that the intervention was received rather than a measure of adherence.

### Visit 4 – Validation of smoking status

This visit will be undertaken either face-to-face or by post, as soon as possible after Visit 3 for all participants who at this visit, report abstinence from smoking for the previous 7 days and the aim is to biochemically validate participants’ reported abstinence. Prior to contacting the participant for their Visit 3 follow-up the Nottingham Trial Office will ask the appropriate recruiting site to ascertain whether the participant will be attending a hospital growth scan at around 36 weeks’ gestation. These scans are used in a number of NHS Trusts for pregnant women who are known to be smoking, or to have smoked prior to their pregnancy. When participants attend hospital for such scans, the Nottingham Trial Office will try to arrange Visit 4, if required, to coincide with this.

Participants will be asked to report their smoking status, recent use of nicotine replacement therapies and/or e-cigarettes. Reported smoking abstinence will be validated using exhaled CO, and/or saliva samples will be taken for cotinine (a nicotine metabolite) [32] and anabasine assays. Anabasine is a tobacco-specific metabolite which reflects tobacco smoke exposure, and so can distinguish this from use of e-cigarettes or nicotine replacement therapy. Exhaled CO will be measured using a validated, hand-held CO monitor and can only be administered by a researcher during a face-to-face visit. Where possible both exhaled CO and the relevant saliva assay will be used together as validation; however, if a valid sample cannot be obtained for one method, the use of the other method alone would be deemed sufficient.

Where it is not possible to arrange a face-to-face validation visit with the participant in either their home or during a hospital visit, a saliva sample kit will be posted to them; they will be asked to provide a saliva sample and to post this back to the Nottingham Trial Office using a secure, pre-paid Royal Mail SafeBox.

Cut-off points for biochemical verification will be determined according to the latest evidence, but defined abstinence is likely to be in the region of < 9 ppm for CO readings and < 10 ng/ml for salivary cotinine [33].

### Duration of the trial

It is anticipated that the total duration of the study will be 36 months. This will include 18 months of recruitment and 9 months of follow-up. Participants of up to 25 weeks’ gestation (24 weeks and 6 days) will be enrolled and late pregnancy outcome ascertainment is intended to be at 36 weeks’ gestation, or at latest, within 2 weeks of birth. However, if outcome data becomes available after this point, we will permit it to be used in analyses provided the timing of data collection is no later than 10 weeks after the estimated due date. Assuming that some individuals might be recruited at their first antenatal visit, at around 10 weeks’ gestation, the maximum possible duration of a participant’s involvement in the trial is, therefore, approximately 40 weeks.

### End of the trial

The end of the trial will be when the late pregnancy outcome has been ascertained for the final participant or it is too long after this participant’s estimated delivery date for such information, if collected, to be used.

### Participant stipends and payments

All participants will receive a £5 high street shopping voucher at each of the four visits to recognise the time taken for their participation in the trial when completing questionnaires. Where all of the first three visits are completed, participants will receive a fourth high street shopping voucher to the value of £10. In addition, a £15 high street shopping voucher will be sent to those participants who provide a saliva sample and/or breath sample during the validation Visit 4 (if required).

### Statistical methods

#### Sample size and justification

We already have data from two very similar RCTs [25, 26] and the study design for this trial is identical to the larger of these [26]. Consequently, it was considered that the most resource-efficient approach would be to complete a third trial which recruits sufficient participants such that when data from all three RCTs is pooled, this gives a definitive answer about the efficacy of the MiQuit service. We pooled data *from the previous trials* and used TSA meta-analysis [34] to estimate that, from the proposed RCT we need an additional 692 participants for a pooled analysis of *all three* RCTs to provide definitive findings regarding effectiveness. However, using pilot study data [26] it is also possible to perform a traditional sample size calculation which determines a 95% confidence interval width which would exclude a minimum important difference between the two intervention groups [35]. Additionally, based on the quit rates from the pilot study (intervention group 5.4% and control group 2%) [26], the proposed RCT with a sample size of 346 participants per treatment arm would, if considered on its own, enable a 95% confidence interval for the difference in quit rates to be estimated to within a precision of ± 3%. Therefore, if this trial observed a 3% difference or greater in quit rates our 95% confidence intervals would not pass through zero.

##### Trial sequential analysis meta-analysis (data synthesis)

Data from this RCT and the two earlier RCTs [25, 26] will be combined in a meta-analysis, using the TSA methods [34] to determine whether or not MiQuit is effective for smoking cessation in pregnancy.

##### Trial analysis (using data from this RCT only):

Analyses, undertaken in Stata v13 or later, will follow the principles of intention-to-treat with outcomes analysed according to randomised groups irrespective of deviations based on non-compliance unless otherwise specified. All outcomes will be analysed once at the trial’s conclusion. Significance tests will be two-sided at the 5% level unless otherwise stated. Parameter estimates will be presented with associated 95% confidence intervals and *p* values as appropriate

*Smoking outcomes*: Where smoking outcome data is missing, we will assume women to be smoking. We will compare smoking outcomes between intervention and usual care groups at the 36-week gestation follow-up point using a penalised logistic regression model adjusting for stratification factors and also potential confounders as fixed-effect covariates. A sensitivity analysis using multiple imputation will assess the robustness of the results to variation in the missing data assumptions.

We will also compare the other smoking-related outcomes between the trial arms using a similar model to the primary analysis. A Complier-Average Causal Effects (CACE) analysis for the primary outcome will be conducted to obtain unbiased estimates of the intervention efficacy with full compliance. Other secondary outcomes will be summarised descriptively by treatment group and comparisons will be made between the groups using appropriate regression techniques.

##### Economic analysis

The economic analysis of the MiQuit 3 trial comprises two components. A ‘within-trial’ incremental cost-effectiveness analysis will be based on an ‘end-of-pregnancy’ horizon using ‘cost-per-quitter’ as an outcome measure. Intervention costs will be prospectively recorded alongside the trial. These include costs of providing the text-message service (staff time, costs of maintaining text-messaging software). Costs of usual care are also recorded as the comparator. The analysis combines intervention costs and wider health care costs with the number of quitters to calculate the cost per quitter of the MiQuit intervention over and above usual care. We will also collect EQ-5D-5 L at baseline and each follow-up to enable to computation of Quality-adjusted Life Years (QALYs) [36].

The longer-term economic evaluation uses a previously developed model [37–39] and pooled efficacy parameters generated by TSA meta-analysis to conduct a cost-utility analysis with maternal and infant lifetime-horizons to estimate the incremental cost per additional QALY. Costs and outcomes will be discounted at 3.5% as recommended by National Institute for Health and Care Excellence (NICE) guidance [40]. We will explore the impact of uncertainty with the use of non-parametric bootstrapping for the ‘within-trial’ analysis and probabilistic sensitivity analysis in the model [41, 42]. This analysis is essential for the MiQuit 3 trial as many adverse health effects of smoking in pregnancy occur beyond the time horizon of final trial follow-up. Long-run cost-effectiveness estimates can be compared with cost per QALY benchmarks to establish the value for money of MiQuit compared to competing claims for health care resources.

### Data management

Data will be entered electronically into a trial-specific database. Only local research staff and the study team at the Nottingham Trial Office will have database access, which permits them to make new entries. Access to participant personal data already recorded on the database will be restricted to Nottingham trial staff and the York Trials Unit. To ensure anonymisation, at randomisation, each participant will be assigned a unique trial identity code number for use on all trial documents and the electronic database. The database will be maintained on a server located within the York Trials Unit, University of York. The database has a regular back-up routine, and will be password-protected. Anonymised data, which are sent to the MiQuit service for tailoring of intervention group participants, will be held on a secure server within the Institute of Public Health at the University of Cambridge. Only authorised staff will have access to trial documentation other than for the regulatory requirements. All trial staff and investigators will endeavour to protect the rights of the trial’s participants to privacy and informed consent, and will adhere to the Data Protection Act, 1998. Electronic data will be backed up every 24 h to both local and remote media in encrypted format.

Monitoring of trial data will include confirmation of informed consent; source data verification; data storage and data transfer procedures; local quality control checks and procedures, back-up and disaster recovery of any local databases and validation of data manipulation. The trial manager, or where required, a nominated designee of the sponsor, will carry out monitoring of trial data as an on-going activity. In compliance with the International Conference on Harmonisation/Good Clinical Practice

(ICH/GCP) guidelines, regulations and in accordance with the University of Nottingham Research Code of Conduct and Research Ethics, the chief or local principal investigator will maintain all records and documents regarding the conduct of the study. These will be retained for at least 7 years or for longer if required. The Trial Master File (TMF) and trial documents held by the chief investigator (CI) on behalf of the sponsor will be finally archived at secure archive facilities at the University of Nottingham.

The study archive will include all trial databases and associated meta-data encryption codes. However, the trial database will be designed by the York Trials Unit using bespoke software, which is not supported outside of the York Trials Unit. Therefore, for data to remain retrievable and potentially useful after the end of the study any data stored in databases created at York will be archived there. The Department of Health Sciences, in which York Trials Unit is based at the University of York, has a back-up procedure approved by auditors for disaster recovery. There will be a separate archival of electronic data performed at the end of the trial to safeguard the data, and in accordance with regulatory requirements.

### Transport, storage and analysis of saliva samples

Saliva samples will be collected from consenting participants who have reported abstinence from smoking, using the methods previously described under Visits 3 and 4. Samples will be obtained using clean salivettes; this involves participants placing sterile swabs under their tongues until moist for up to 5 min and then placing the swab into a sterile vessel. Samples will be labelled and held within Nottingham Health Science Biobank (NHSB) in a secure freezer storage unit (− 80 °C) according to approved protocols. Where local research staff obtain samples, these will be posted by the study team in suitable packaging, and when received, the study team will transfer them to NHSB. Saliva and anabasine samples are stable at ambient temperatures for several days. Nottingham Health Science Biobank has been given full approval by the Human Tissue Authority (HTA) to be a full licence holder, meeting all legislation requirements.

NHSB will arrange transportation of samples by courier to ABS Laboratories Ltd., Hertfordshire, UK for analysis in a single-batch shipment. The shipment will contain a complete inventory of all samples, along with the name of the person responsible for sending the samples. The master database to link all samples will be held by the Nottingham study team in a password-encrypted file. The laboratory will estimate salivary cotinine and anabasine levels using a quantitative enzyme immunoassay technique (EIA).

Once the analysis has been completed the saliva samples will be destroyed in accordance with the Human Tissue Act 2004, this will only occur once the study team has received the results and has analysed the data to ensure that all samples remain in a normal range and do not require retesting.

### Dissemination

Results will be written up for publication in peer-reviewed journals and disseminated at local, national and international meetings where appropriate. Papers describing the key findings will be submitted within 12 months of the trial completion. A lay summary will be produced and distributed to those participants who have indicated that they would like to receive a copy, and other interested parties. Participants will not be identified in any publications or presentations resulting from this study.

### User and public involvement

A Public and Patient Involvement (PPI) representative has contributed to this protocol, to trial documents and to the development of the intervention.

### Indemnity

Insurance and indemnity for trial participants and trial staff is covered within the NHS Indemnity Arrangements for clinical negligence claims in the NHS, issued under cover of Health Service Guidelines (HSG) (96)48. There are no special compensation arrangements, but trial participants may have recourse through the NHS complaints procedures.

The University of Nottingham as research sponsor indemnifies its staff, research participants and research protocols with both public liability insurance and clinical trials insurance. These policies include provision for indemnity in the event of a successful litigious claim for proven non-negligent harm.

### Trial management

The CI has overall responsibility for the study and will oversee all study management. The Trial Management Group (TMG) will be responsible for the day-to-day running of the trial. The TMG will meet on a monthly basis and will be supported by and reporting to a Trial Steering Committee (TSC), which will meet as and when required; a separate Data Monitoring and Ethics Committee is not judged necessary, as we cannot envisage the intervention having the potential to harm participants. The names of TSC members and their roles will be stated on the University of Nottingham’s TSC Charter (v1.2 October 2014) and the TSC Membership Agreement (v1.1 October 2014).

Trial co-ordination, with respect to recruitment and follow-up will be managed centrally by the study team based in Nottingham, led by a trial manager and guided by the TMG.

Trial co-ordination, with respect to data management and analyses will be managed by the York Trials Unit team, again guided by the TMG. The Nottingham trial manager will be responsible for overall co-ordination across the lead sites for management of recruitment (Nottingham) and data (York).

The Cambridge research team will manage a server hosting the MiQuit intervention; however, the data custodian will be the CI.

### Sponsor

The trial is sponsored by the University of Nottingham.

## Discussion

Once completed, the results from this definitive trial should provide sufficient data to determine, using Trial Sequential Analysis (TSA) meta-analysis, whether or not MiQuit shows efficacy when offered in addition to standard behavioural support for smoking cessation in pregnancy. TSA methods have previously been used to assess the likelihood that positive or negative findings from traditional meta-analyses are valid and we believe that our proposed use of TSA meta-analysis methods is novel.

TSA is a method for pooling RCT data which quantifies the statistical reliability of data in a cumulative meta-analysis adjusting significance levels on accumulating data for small numbers and repetitive testing [34]. This method is useful for assessing intervention efficacy when few, relatively small trials are available. We have data from two smaller trials which randomised 614 participants, [25, 26] and combining data from the additional 692 participants recruited in this RCT with these previously collected data in a TSA meta-analysis will derive a pooled estimate for MiQuit efficacy. This third RCT has an almost identical design and outcomes to earlier ones; if there were substantial differences in trial protocols or outcomes, TSA meta-analysis might not have been possible. Additionally, this approach might not have been appropriate if the intervention was potentially harmful to women or their babies because, in that instance either a full trial or a TSA meta-analysis might have needed sufficient power to also detect whether or not MiQuit caused adverse outcomes. However, it is difficult to foresee how MiQuit could be harmful and so this does not apply.

Apart from using other forms of text-message support, MiQuit 3 trial participants will be free to access whatever NHS support with stopping smoking is available in their locality. As such, if MiQuit does prove to have a positive effect on cessation, this will be additional to that which participants have obtained via usual NHS means and so, adjunctive to standard smoking cessation support provided during pregnancy. Therefore, if it is proven to work, MiQuit could be disseminated to pregnant smokers either alongside usual stop-smoking support or in parallel to this in the expectation of it having positive effects on women’s smoking. This would also be relatively easy as MiQuit is fully adapted for ‘self-initiation’ and users can activate the service after finding it on the web [43], or after seeing a leaflet [44]. Additionally, MiQuit is such a cheap intervention that if it has even a very small impact on cessation it could prove very cost-effective and so the proposed, longer-term, model-based economic evaluation which will follow the RCT is likely to be of particular interest to policy-makers (Additional file 1).

### Trial status

Recruitment began in November 2017, with the first participant randomised on 4 December 2017. At the time of the manuscript submission, the trial was still recruiting with 455 participants recruited by 27 April 2018. It is anticipated that recruitment will be completed by September 2018. This article is based on protocol version 1.1, 29 September 2017.

## Additional file

Additional file 1: Standard Protocol Items: Recommendations for Clinical Trials (SPIRIT) 2013 Checklist: recommended items to address in a clinical trial protocol and related documents*. (DOC 120 kb)

## Abbreviations

BHF: British Heart Foundation
CACE: Complier-Average Causal Effects
CI: Chief investigator
CO: Carbon monoxide
CRUK: Cancer Research UK
DHSC: Department of Health and Social Care
EIA: Enzyme immunoassay technique
EM CLARHC: East Midlands Collaboration for Leadership in Applied Health Research and Care
EQ-5D-5 L: EuroQol-5D-5 Level Questionnaire
ESRC: Economic and Social Research Council
HSG: Health Service Guidelines
HTA: Human Tissue Authority
ICH/GCP: International Conference on Harmonisation/Good Clinical Practice
MRC: Medical Research Council
NHS: National Health Service
NHSB: Nottingham Health Science Biobank
NICE: The National Institute for Health and Care Excellence
NIHR: National Institute for Health Research
NRES: National Research Ethics Service
NRT: Nicotine replacement therapy
PFL: Pierre Fabre Laboratories
PIL: Participant Information Leaflet
PPI: Public and Patient Involvement
QALYs: Quality-adjusted Life Years
RCT: Randomised controlled trial
SHS: Self-help support
SPCR: School for Primary Care Research
TMF: Trial Master File
TMG: Trial Management Group
TSA: Trial Sequential Analysis
TSC: Trial Steering Committee
UKCTAS: UK Centre for Tobacco and Alcohol Studies
